# The Young and Poor

**Published:** 1860-12

**Authors:** 


### THE YOUNG AND POOR.

To be young is glorious; to be poor and young, with a will to excel, is one of the greatest blessings which can befall a youth, as it is a certain curse, and a deep one, to be young and rich, and have no ambition but to lounge about, with the only aim of whiling away the time until the death of parents puts them in possession of inherited treasures. There are three books which merit a place in every public library, and which would be of inestimable value to any family of children, and to any youth who has the courage and energy to achieve a name or a position: *Smiles' Self-Help*, 75 cents; *Life of George Stephenson*, $1; *Brief Biographies*, $1.25. They show, by simple facts, in plain and vigorous language, what a multitude of men were as boys, poor, without influence, and in cases not a few, without friends who had the ability to give them the slightest aid. Let every parent who is anxious that his child should live to purpose, and who would place before him the high stimulus of honorable example to that same end, give that child these three books, opportunely published, in inviting style, by the Messrs. Ticknor & Fields. They can be had at any first-class book-store.

